# Investigation of the key factors that influence the girls to enter into child marriage: A meta-synthesis of qualitative evidence

**DOI:** 10.1371/journal.pone.0235959

**Published:** 2020-07-17

**Authors:** Ayako Kohno, Teeranee Techasrivichien, S. Pilar Suguimoto, Maznah Dahlui, Nik Daliana Nik Farid, Takeo Nakayama

**Affiliations:** 1 Internationalization Promotion Office, School of Public Health, Graduate School of Medicine, Kyoto University, Kyoto, Japan; 2 Department of Health Informatics, School of Public Health, Graduate School of Medicine, Kyoto University, Kyoto, Japan; 3 Interdisciplinary Unit for Global Health, Center for the Promotion of Interdisciplinary Education and Research, Kyoto University, Kyoto, Japan; 4 International Institute of Socio-Epidemiology, Kyoto, Japan; 5 Medical Education Center, Graduate School of Medicine, Kyoto University, Kyoto, Japan; 6 Centre for Population Health, Faculty of Medicine, University of Malaya, Kuala Lumpur, Malaysia; 7 Department of Health Policy and Administration, Faculty of Public Health, Airlangga University, Surabaya, Indonesia; 8 Department of Social and Preventive Medicine, Faculty of Medicine, University of Malaya, Kuala Lumpur, Malaysia; Coventry University, UNITED KINGDOM

## Abstract

In this study, we synthesized findings from qualitative studies to identify the key factors that influence child marriage. We used a meta-ethnographic approach coupled with thematic synthesis. We searched literature from nine databases, which were in English language, covering areas in public health, psychology, and social science between 2008 and 2018. Twelve studies were included in the synthesis. We identified six main themes: human insecurity and conflict; legal issues; family values and circumstances; religious beliefs; individual circumstances, beliefs, and knowledge; and social norms. Our findings highlight the impact of human insecurity and conflict, as well as legal issues. In spite of global progress scaling up legislation against child marriage, the legal framework is insufficiently enforced in many settings. Most of the included studies were from the Middle East, Africa, and South Asia. Studies from other parts of the world such as Latin America and Southeast Asia, which have the highest rates of child marriage, are needed.

## Introduction

Child marriage refers to formal or informal marriage that involves children who are below the age of 18 [1]. Although the term applies to both boys and girls, girls are the most commonly affected by this practice [2]. It is a harmful practice that violates children’s rights; unfortunately, it remains widespread. The UNICEF reported a reduction in the global prevalence of child marriage in the last decade, which was largely driven by prevention and care interventions in South Asia. However, 12 million girls per year still get married before the age of 18 [3]. Thus, we have approximately 650 million women today who were married as children. The highest levels of child marriage are found in Sub-Saharan Africa (38%), with Niger reporting a prevalence as high as 76%, followed by South Asia (30%) and Latin America (25%) [3]. According to data from the Demographic and Health Surveys (DHS) in Bangladesh, 59% and 22% of women were married before the age of 18 and before the age of 15, respectively. In Latin America, the numbers are also alarming. In the Dominican Republic and Brazil, 36% of women were married before the age of 18 [4].

There are some theories in cross-cultural perspectives that are relevant to the investigation of factors that influence child-marriage. Kagitcibasi’s theory of family change suggests the link between sociocultural factors and development of the self, and family as a mediator [5]. This model describes the relationship between society/culture, family, and the resultant self, as well as how children are perceived as economic value by the parents. Additionally, we adopted a theory by Gelfand et al. where they explained the concept of cultural tightness-looseness that portrays the role of social norms that determines the sociocultural sanctions imposed on people within the societies [6]. In this study, we examined the factors that influence child marriage by using these theories as the conceptual frameworks.

Meta-ethnography is an approach that synthesizes qualitative studies. It consist of seven phases; identifying an intellectual interest, deciding what is relevant, reading the studies, determining how the studies are related, translating the studies into one another, synthesizing translations, and expressing the synthesis [7]. This method was developed by Noblit and Hare in 1988. This method was employed in this study, in order to provide interpretive explanation by integrating the results of the qualitative studies. While findings of quantitative studies can provide results in terms of the association of some risk factors for child marriage, they cannot describe the subtle nuance of the interpretation of people’s perception of these risk factors. Therefore, we believe that this study complements the existing knowledge regarding child marriage by providing synthesized qualitative insights.

Child marriage often results in adverse health, economic, and social consequences [8]. Catalyzed by socio-economic imbalance, spousal age gap, power imbalance, social isolation, and lack of female autonomy, studies show a significant association between child marriage and all dimensions of intimate partner violence (physical, sexual, and emotional) [9–14]. For girls, child marriage translates into early sexual activity. They are more likely to report no contraceptive use and are at a greater risk of unintended pregnancy [8] and sexually transmitted infections including HIV and cervical cancer [15]. Among women aged 15–19 years, pregnancy-related death is the second leading cause of death [16]. Physical immaturity for childbearing, combined with lack of power, information, and access to services, place them at a heightened risk of maternal morbidity and mortality. However, the practice of child marriage not only affects the girls who are getting married at a young age, but also affects the girls’ offspring. This has been associated with adverse child outcomes including preterm birth and intrauterine growth restriction, infant mortality, and child undernutrition [16]. A study using data from 37,558 mother-child pairs pooled from 16 national and sub-national surveys across sub-Saharan Africa (2010–2014) examined the relationship between girl-child marriage and the development of their children early in life. Results show that children born to women who were married before the age of 18 had 25% higher odds of being off-track in terms of development and 29% higher odds of experiencing stunted growth compared to those whose mothers married later [17]. This was explained by the disparities in advanced maternal education and wealth. The relationship between child marriage and maternal and infant mortality is well documented at the global level [18].

The devastating consequences of child marriage, and the negative impact of this practice on achieving development have been recognized by its inclusion in Goal 5 of the sustainable development goals (SDGs) [19]. Evidence suggests that the determinants of child marriage vary greatly depending on socioeconomic status, region, culture, and religion [20–22]. Despite the global significance of this harmful practice, to the best of our knowledge, there are no previous systematic reviews of qualitative findings exploring the factors behind child marriage. Quantitative survey data provide important information on trends and associations between child marriage and sociodemographic characteristics. However, they only present a partial picture of the factors that influence child marriage [23]. We, therefore, conducted a meta-synthesis to fill this gap. The aim of the study is to identify the key factors that influence child marriage, and synthesize the findings obtained from qualitative studies.

## Methods

We applied a thematic synthesis methodology [24] which consisted of three steps: coding of text “line-by-line,” developing “descriptive themes,” and generating “analytical themes.” We selected this methodology because we wanted to 1) provide additional insights by “going beyond” the mere integration of the findings of multiple qualitative studies, 2) utilize thematic analysis as the basis of synthesizing the results, and 3) identify the key concepts from the primary qualitative research and translate them into one another. To ensure that the procedures of this meta-synthesis were valid and standardized, we used a meta-ethnographic approach [7].

### Inclusion and exclusion criteria

We considered qualitative studies that meet the following inclusion criteria:

1. Focus on the lived experiences of women who were married before the age of 18, and the perspectives of the stakeholders.
2. Using data collection methods such as interviews, focus group discussions, case studies, or observations.
3. Clearly state that the authors used qualitative methodology in the analysis.
4. Published in English language.
5. Published between Jan 1, 2008 and December 31, 2018.

Criteria for exclusion were:

1. Mixed method studies or studies including quantitative data.
2. Focus on maternal care and care of the newborn.
3. Commentary articles.

The mixed method studies were not included in this synthesis, as it is difficult to distinguish what were the qualitative findings from quantitative results arising from a mixed-method study.

### Literature search strategy and retrieval of studies

We conducted a literature search using nine electronic databases including PubMed, PsycINFO, CINAHL, Scopus, ProQuest, Web of Science Core Collection, ScienceDirect, Kyoto University Discovery and Google Scholar. The search was conducted between April 2017 and December 2018. Key search words included: “women,” “girl,” “young*,” “teen,” “mother,” “child marriage,” “early marriage,” “teenage marriage,” “health,” “interview,” “focus group*,” “case stud*,” “observ*,” “view*,” “experience*,” “opinion*,” “attitude*,” “percep*,” “belie*,” “feel*,” “know*,” “understand*,” “qualitative.” We combined key words by applying the Boolean operators AND/OR.

**Fig 1:**
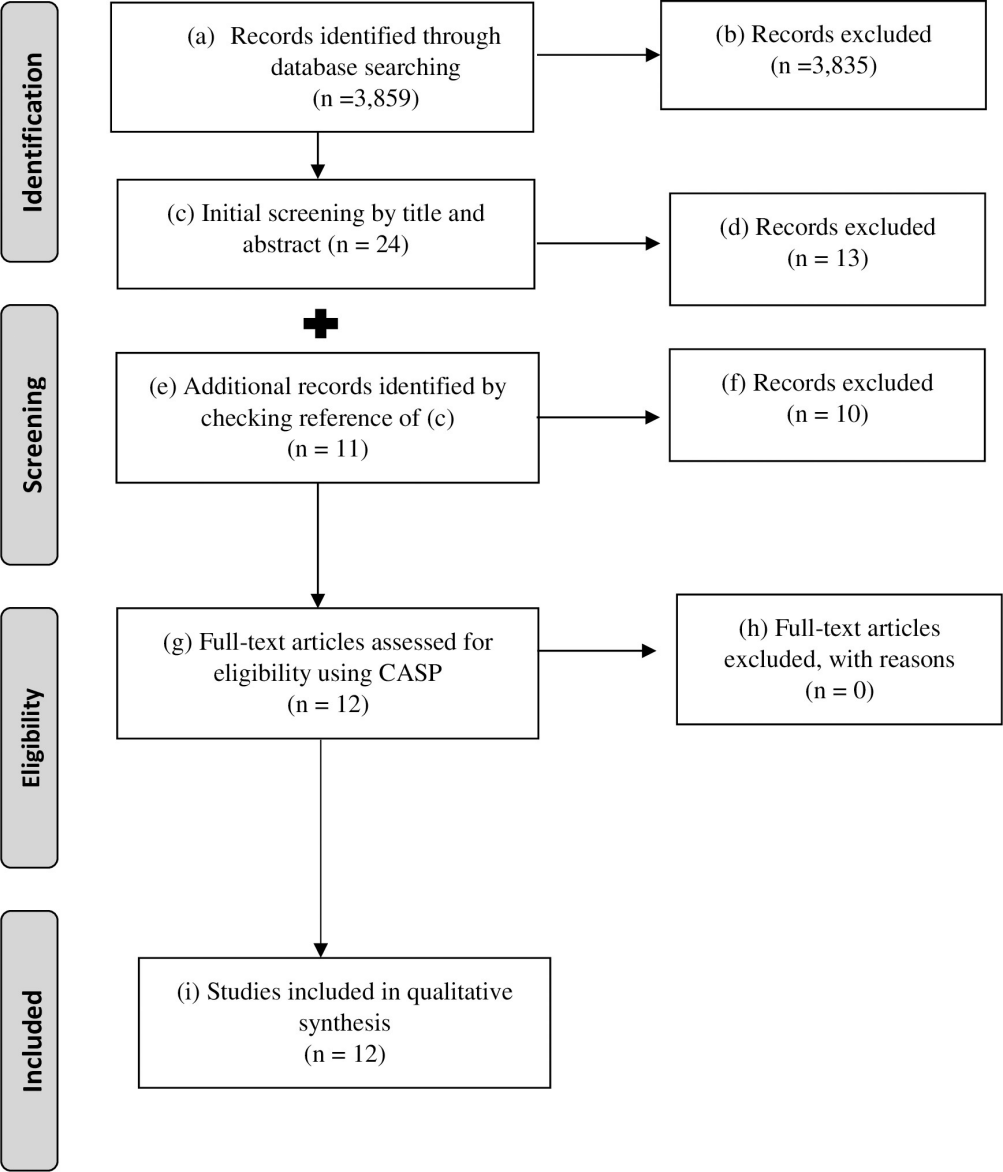
Search strategy and outcome.

We screened all titles and abstracts of the initial 3,859 “hits,” and after eliminating duplicates and those not satisfying the inclusion criteria (3,835 articles), we filtered 24 potentially eligible studies. We checked their reference lists and identified 11 further studies. Upon reading the articles in full text, we finally selected 12 studies for our analysis. Fig 1 shows the overall process.

### Appraisal of selected studies

We assessed the quality of the 12 selected studies using the Critical Appraisal Skills Programme (CASP) for qualitative research [25]. This is a widely used tool that consists of a 10-question checklist that is comprehensive in its scope, and is easy to understand and administer. Most questions are answered with “yes,” “no,” or “can’t tell.” The first two questions are for screening purposes, asking about the aim of the study and the appropriateness of the methodology to the aim. If the answer to both questions is “yes,” then it is worth asking the remaining 8 questions about appraisal of the study (research design, recruitment strategy, data collection, reflexivity, ethics, rigor of data analysis, and clear reporting of findings and its implications).

Three researchers (AK, TT, and SPS) independently read, reread, and appraised the articles. A three-point rating system developed by Duggleby and colleagues was adopted in this study to quantify the appraisal from the CASP checklist and obtain an overall score of quality [26]. Each of the eight questions (appraisal questions) in the CASP checklist were assigned one to three points, giving each study a maximum possible score of 24. Studies offering minimal to no justification or explanation for a particular issue were given 1 point; those addressing but not fully elaborating the issue were given 2 points; and studies that extensively justified and explained the issue at hand were given 3 points (see Table 1). Studies were not excluded based on the CASP score. We discussed the results of our quality appraisal through regular meetings, resolved discrepancies by reflecting, and decided on the final 12 articles until agreement was reached through consensus. We observed a variation of the scores for some of the CASP questions, such as “Consideration of Researcher and Participants Relationship” (see Table 1). This is due to the fact that qualitative research focusing on child marriage is still scarce and we decided to include broadly as long as the studies at least fulfills the basic requirements of what is listed in CASP. We admit that there is a diversity in the quality of the included studies in terms of the particular viewpoint of description about the relationship between the authors and the participants of each study. However, our purpose was to conduct initial investigation of what can be revealed by including the currently available qualitative publications on child marriage. Therefore, we did not adopt the inter-rater reliability approach for the evaluation in using the CASP checklist.

**Table 1:** Quality appraisal of the reviewed articles.

	CASP											
	3 point-rating system*											
No.	Main Author	Clear Aim	Appropriate Methodology	Research Design	Recruitment Strategy	Data Collection	Consideration of Researcher and Participants Relationship	Ethics	Data Analysis	Findings	Implications	Total Score
1	Callaghan	Yes	Yes	3	3	3	3	3	3	3	3	24
2	Cherri	Yes	Yes	3	3	3	1	3	3	3	3	22
3	Mangeli	Yes	Yes	3	3	3	2	3	3	2	3	22
4	Montazeri	Yes	Yes	3	3	3	2	3	3	3	3	23
5	Mourtada	Yes	Yes	3	3	3	2	2	3	3	2	21
6	Nasrullah	Yes	Yes	3	3	3	3	3	1	1	3	20
7	Sabbe	Yes	Yes	3	3	3	3	3	3	3	3	24
8	Sabbe	Yes	Yes	3	3	3	3	3	3	3	3	24
9	Vang	Yes	Yes	3	3	3	2	3	3	3	3	23
10	Mardi	Yes	Yes	3	3	3	3	3	3	3	3	24
11	Raj	Yes	Yes	3	3	3	2	3	3	3	3	23
12	Knox	Yes	Yes	3	3	3	3	3	2	3	3	23

*1 point: offers little to no justification or explanation for particular issues (when, where, or how). 2 points: addresses the issue but not fully elaborates on it. 3 points: extensively justifies and explains the issue at hand.

### Analysis, synthesis, and reporting

In the analysis of the synthesized studies, the steps of thematic synthesis were followed as explained above. First, entire sections of each of the included studies were read and reread by three authors (AK, TT, and SPS) in order to familiarize with the contents of each article. Subsequently, the text from the results and discussion sections of the included articles were coded inductively and individually “line-by-line” to derive initial codes. In doing so, we categorized the codes into three groups: “first-order” theme representing the perspectives and understanding of the participants with regards to the studies; “second-order” theme representing the primary authors’ interpretations; and “third-order” themes reflecting the researchers’ interpretations (AK, TT, and SPS). Afterwards, based on the created codes, we developed “descriptive themes” that articulated the holistic perspective of multiple studies by comparing the codes developed for each study. Finally, we generated “analytical themes” by clustering and synthesizing the descriptive themes, and added our original interpretation. Microsoft Excel software was used for data management and analysis. Additionally, a qualitative metasummary was adopted as a way to visually represent the findings of this study by calculating the effect size [27]. For the reports, we followed the guideline of Enhancing Transparency in Reporting the Synthesis of Qualitative Research (ENTREQ) [28].

## Results

Twelve articles were included in this meta-synthesis; 6 of which were conducted in the Middle East (Lebanon and Iran), 3 in Africa (Nigeria and Morocco), 2 in South Asia (Pakistan and Afghanistan), and 1 in the United States. Sample sizes ranged from 6 to 125 (overall, approximately 550 participants); subjects included women who were married before the age of 18; unmarried, divorced, or engaged adult women; and various stakeholders. The most commonly adopted method of data collection was individual interviews (in-depth, semi-structured, or in-depth semi-structured). Table 2 shows the basic demographic and methodological characteristics of all included articles, and Table 3 shows the metasummary of included studies with effect size for each theme to express the frequency of the themes.

**Table 2:** Basic demographic and methodological characteristics of included articles.

No.	Authors (Year)	Place of study	Participants	Age of women interviewed (group mean)	Women’s age at marriage (mean)	Sample Size	Research Design	Data Collection
1	Callaghan et al. (2015)	Nigeria (Sokoto state)	Nigerian married women	25–35	8–15	6	Interpretive phenomenological analysis	Individual interviews
2	Cherri et al. (2017)	Lebanon (Beirut and Mount Lebanon, South, North, and Bekaa)	Syrian refugee women (married and unmarried)	15–49	no data	108	Thematic analysis	FGDs
3	Mangeli et al. (2017)	Iran (Kerman province)	Iranian mothers	adolescents	12–17 (15.4)	16	Content analysis	ID-SSIs
4	Montazeri et al. (2017)	Iran (Ahvaz)	Iranian married women	<19	no data	15	Content analysis	ID-SSIs
5	Mourtada et al. (2017)	Lebanon (Al Marj in Bekaa area)	Syrian refugees (married women, unmarried women, and their parents), and stakeholders (NGO and government officials, camp leader, religious leaders, teacher and gynecologist)	18–24	<18	61	Thematic analysis	FGDs, SSIs
6	Nasrullah et al. (2014)	Pakistan (slums of Lahore city)	Pakistani married women	21–34	11–17	19	Qualitative study	IDIs
7	Sabbe et al. (2015)	Morocco (Marrakech region)	Moroccan women (married and unmarried)	18–69	no data^1^	125	Thematic analysis	SSIs and FGDs
8	Sabbe et al. (2013)	Morocco (Rabat, Casablanca, and Marrakech region)	Moroccan stakeholders (teachers, academics, healthcare workers, lawyers, government and NGO representatives)	-	-	22	Thematic analysis	SSIs
9	Vang & Her (2014)	United States of America	Hmong American married women (US, Laos, and Thailand born)	22–39	14–17	12	Qualitative study	SSIs
10	Mardi et al. (2018)	Iran (health-care centers in Ardabil)	Iranian married women	12–18 (14.9)	12–16 (13.2)	14	Content analysis	ID-SSIs
11	Raj et al. (2014)	Afghanistan (Kabul, Jalalabad, Masar)	Afghan stakeholders (religious leaders, police, teachers, NGO and government officials)	-	-	112	Grounded theory approach	IDIs and group interviews
12	Knox (2017)	Lebanon (Palestinian refugee camp)	Palestinian women refugees (married, divorced or formally engaged), and stakeholders (women’ mothers, NGO workers)	Adolescent	Under 18	54	Thematic content analysis	IDIs and FGDs

FGDs: Focus group discussions; ID-SSIs: In-depth semi-structured interviews; SSIs: Semi-structured interviews; IDIs: In-depth interviews; -: not applicable.

^1^, 40% of the sample was unmarried or single.

**Table 3:** Metasummary table.

Themes	1	2	3	4	5	6	7	8	9	10	11	12	Effect Size
	Callaghan	Cherri	Mangeli	Montazeri	Mourtada	Nasrullah	Sabbe	Sabbe	Vang	Mardi	Raj	Knox	
	2015	2017	2017	2016	2017	2014	2015	2013	2015	2018	2014	2017	
Human Insecurity and Conflict		X	X		X	X	X	X		X	X	X	0.75
Legal Issue					X		X	X			X		0.33
Family Values and Circumstances	X		X	X	X	X	X	X	X	X	X	X	0.92
Religious Belief		X	X	X	X	X	X	X		X	X		0.75
Individual Circumstances, Beliefs and Knowledge	X			X			X	X		X	X	X	0.58
Social Norms	X	X	X	X	X	X	X	X	X		X	X	0.92

### Theme 1: Human insecurity and conflict

The practice of child marriage was exacerbated by human insecurity and conflict. This theme appeared in nine of the 12 studies [29–37]. In these studies, the participants (refugee women (married and unmarried), mothers who experienced child marriage, stakeholders (NGO and government officials, camp leader, religious leaders, teacher, gynecologist, academics, police and lawyers)) described experiencing physical and emotional insecurity, either as refugees, or when living in slums or post-conflict settings. Several subthemes emerged in this theme:

#### Feelings of insecurity

In the case of Syrian refugees in Lebanon, many of the participants, parents and adolescent girls, reported feelings of insecurity as they experienced verbal or physical harassment while residing in refugee settlements: “Fear of insecurity is a major factor. They are marrying early because of al Sutra. We have war. Many women are afraid of being raped, and if a married woman is raped, she is more likely to be forgiven by her husband but if an unmarried woman is raped, it will destroy her life” [31]. In the case of parents, this fear was the driving force to marry off their daughters. Parents living in situations of conflict and displacement had concerns about the safety of their daughters’ chastity, and felt that the girls would be safer if they were married early.

Security concerns were evident both in the home country and in the settlements. There were reports that parents were purposefully sending their daughters from areas of conflict in Syria to get married at a young age in Lebanon, in order to escape the conflict and provide protection for their daughters. Parents were worried that their daughters may experience physical attacks or rape before getting married in Syria, which would not only harm the girls but also hinder their future: “Moreover, they [unmarried adolescent women] were also vulnerable to sexual harassment, and occasionally even rape due to living in unfamiliar and sometimes insecure areas and the breakdown of social networks” [31]. In such cases of marriage in the settlements, the daughters who were getting married had never seen the future husbands before the day when they got married.

#### Financial constraints

Another factor causing insecurity among refugee parents or those living in the slums that encouraged early marriage was financial constraints. In the midst of the conflict, families in displaced settlements tended to be of large numbers and parents sought to relieve their responsibilities and reduce the family’s financial burden by letting their daughters get married at an early age: “Some people marry off their daughters at an early age because these are their traditions but others do it to ease the financial pressure. For example, there are fathers who are jobless and who have to provide for 5 or 6 people, so marrying off one of their daughters will help” [31]. This is a view shared by some adolescent girls who agreed to their parents’ wishes. Other girls who lived in constrained spaces with numerous family members crammed into small rooms viewed marriage as an escape from their situation. In the case of those residing in slum areas in Pakistan, it was evident that child marriage would be favored by women from poor, low-educated families in the rural areas: “Based on the occupational categories of the respondents and their husbands, fifteen respondents (78.4%) belonged to a low socio-economic class” [32]. The parents perceived that the girls would become the responsibility of their husbands who would then protect and take care of them financially.

#### Fear of losing the opportunity

For adolescent girls, the fear of not being able to find a suitable partner was a motivation to accept early marriage: “I knew him very well. They were very nice people… he met my criteria. I was going to school that time, but I thought, if I got married I would be better off because my husband had a good condition. I did not want to miss the chance.” Their idea of a perfect suitor was someone with a good personality, whether they know the family of the future husband or not. Further, there was a prevailing belief in the society under study, such as in southeast province in Iran and suburban area in Morocco, that when a girl’s age increases, her opportunities for marriage would diminish. This belief was prominent in the traditional Iranian culture, especially in the rural areas. Thus, it acted as pressure pushing the young unmarried girls to accept to get married at a young age at their first suitable opportunity.

### Theme 2: Legal issues

Among the 12 articles, legal issues emerged from four studies [31, 33, 34, 36] as a push factor for child marriage. The participants (refugees (married women, unmarried women, and their parents), women (married and unmarried) and stakeholders (NGO and government officials, camp leader, religious leaders, teachers, academics, healthcare workers, lawyers, police and gynecologist)) described how the legal issues are accelerating child marriage.

#### Insufficient legal protection

In Morocco, despite the introduction of the new Family Code (Moudawana) in 2004 raising the minimum age for marriage from 15 to 18 years of age, there is an increase in the number of young women forced into marriage. There were 41,098 child marriages authorized in 2010, an increase of 23.6% from 2009. Sabbe et al. explored women’s perspectives on marriage and rights in Morocco in relation to the factors that contribute to child and forced marriages [33]. The findings demonstrated that there was an overall agreement among the study participants that there are loopholes in the existing legal framework, and often, these legislations are not being enforced properly. The participants reported that cases of corruption around marriage were common; for example, some institutions that conduct wedding ceremonies could be bribed when the young women were below the legal minimum age: “There are ways to circumvent the law … Institutions that conduct the wedding ceremony are bribed in order for marriage to take place at a younger age” [33].

#### Legal and social divergence

Sabbe et al. interviewed a broad range of stakeholders in Morocco about their perspectives on factors that contribute to child and forced marriage [34]. Out of the major themes that emerged around legal issues, “legal and social divergence in conceptualizing forced and child marriage” was included. In many circumstances, it was difficult to judge whether a girl agreed to the union out of free will. There were clear-cut cases of physical violence; however, emotional or mental pressure from the family might lead to feelings of anxiety and fear that could overrule the girl’s resistance to marry: “There is pressure, often from the family, that is a given. But once there is a signature, suggesting an agreement to the marriage, the contract is valid. Without consent the contract is void, and therefore the marriage has never taken place” [34]. Moreover, some participants reported situations in which the family court judge arbitrarily authorized the underage marriage, whereby, appearing to protect the young girls from stigma stemming from the loss of virginity or pregnancy, or to provide them the opportunity of a “better” life abroad. Participants acknowledged the so-called “legal pragmatism” faced by judges. Their ruling was often inconsistent with the law and biased in favor of approving child marriage because their social expectations and patriarchal vision of the family unit influenced their decisions. Another important theme found by Sabbe et al. was the “impact of legislation”, where discussions predominantly evolved around the “Rape Marriage Law.” To circumvent sexual relations outside of marriage, Article 475 of the Moroccan Penal Code states that the abductor of a minor (without using violence, threat or fraud) can escape prosecution and imprisonment by marrying the victim. Participants cited this as “Institutional rape.” Although Article 475 was amended in 2014, and the aforementioned statement about marriage was eliminated from the Moroccan Penal Code, it affected many girls who had been married due to rape prior to this year in Morocco.

#### Participants’ knowledge related to legal age for marriage

Raj et al. identified perspectives on the causes of and potential solutions to child and forced marriage among religious leaders, the police, teachers, and nongovernmental organizations (NGO) and government officials in Kabul, Jalalabad, and Mazar, Afghanistan [36]. Regarding the participants’ knowledge and perspectives related to the legal age for marriage in Afghanistan, participants were aware of the legal age for marriage (16 years). However, most participants felt that the legal age for marriage should be raised to 18 years. In contrast, for religious leaders, an older age for marriage among women is not in line with Islamic law which considers that reaching puberty is appropriate for marriage, hence, 16 years is a suitable age if the girl has begun experiencing signs of puberty: “Islamic and Sharia law states that a girl should reach puberty before marriage, so 16 years is appropriate provided the girl’s puberty has begun” (male religious leader) [36]. Nonetheless, most religious leaders described child marriage (below the age of 16) and forced marriage as “haram” (against God) based on the Quran which states that a marriage requires both adulthood and consent from the bride and groom. In their opinion, however, adulthood was not necessarily consistent with the legal age as stipulated by the law, as they consider that some women are already mature from the age of 12.

### Theme 3: Family values and circumstances

Among the 12 studies, “family values and circumstances” emerged from 11 studies as a determinant for child marriage [30–40]. The participants (women (married and unmarried), mothers who experienced child marriage, refugees (married women, unmarried women, and their parents), and stakeholders (NGO and government officials, camp leader, religious leaders, teachers, academics, healthcare workers, lawyers, police and gynecologist)) told how the family values and circumstances were affecting the decision of child marriage.

#### Marrying-out away from family

Mangeli et al. explored the perspectives of adolescent mothers in Kerman, Iran, with regards to factors that encouraged them to get married at an early age [30]. “Instability within the family,” in particular, family breakdown and divorce or death of parents, were cited as causes of early marriage. Participants viewed that “marrying-out” away from family would lead to a better situation; i.e., one participant was unable to get along with the new husband of her mother, and ‘marrying out’ was perceived as the best solution: “I was nine years old when my parents got divorced… my mother married another man. I had a lot of problems with my stepfather and half-sisters and brothers. I could not accept my stepfather as my father” [30]. Furthermore, some adolescents got married at an early age due to the “Desire and encouragement of parents,” which in turn, may have been largely influenced by financial problems, social norms, and cultural and religious issues.

#### Cultural family values normalizing child marriage

Montazeri et al. interviewed women who visited the health care centers in Ahvaz, Iran, in an effort to understand the determinants of early marriage [38]. “Cultural family values” emerged as a facilitator for child marriage. In their studies, the parents of girls inculcated in their daughters that early marriage is a desirable thing to pursue [30, 38]. In Morocco, grandparents and older relatives have a strong influence on household decisions including the marriage of the young member of their family: “The stance of these women essentially suggests that potential victims of a forced marriage should let themselves be convinced by their fathers or older relatives to go ahead with the unwelcome marriage and, above all, convince themselves that they actually want to marry the proposed partner” [33, 34]. Parents and grandparents teach the adolescent girls that marriage at an early age is virtuous. From the participants’ perspectives, being encouraged by family to get married greatly influenced their decision making. Mangeli et al. described that, as the Iranian mothers preferred that their daughters get married at the same age as they did, it is difficult to alter the vicious cycle of child marriage through generations in the family [30].

Furthermore, the quality of the suitor seemed to have played a role in the parents urging their children to get married. Parents would encourage their daughter, regardless of her age, to marry a “suitable” candidate who is well educated and of good moral and economical status: “Regardless of girl’ age, if any suitor was morally and economically in good condition, parents would encourage their daughter to marry him: …‘The boy was polite, educated and had a good job. He also had no problem with me to continue my education after getting married. My mom wanted me to marry him’” [38]. Further, consanguineous marriage was the most preferable. However, if a suitor among the relatives was not viewed as appropriate, parents would reject him, which reflects that parents do play a major role in approving a marriage. Vang et al. discussed the insights of Hmong American women who were married under the age of 18 with regards to their perspectives related to their early marriages. Teenage marriage is internalized as a family discourse and cultural norm in a “family culture normalizing teenage marriage.” Family members constantly and commonly conveyed the acceptability and preference of teenage marriage through family stories and in everyday conversation, thus, exposing the participants to the notion of marriage at an early age: “When I was younger, I remember my parents always telling me that I had to learn how to be a good wife and they used to say things like ‘if you don’t get married when you’re still young, no one will want to marry you when you’re old.” [39].

### Theme 4: Religious beliefs

The theme of religious beliefs appeared in nine of the 12 studies [29–36, 38]. The influence of religion was strong in most of the studies. In this study, we observed that the participants (refugee women (married and unmarried), mothers who experienced child marriage, their parents, and stakeholders (NGO and government officials, camp leader, religious leaders, teachers, academics, healthcare workers, lawyers, police and gynecologist)) referred to Islamic religious beliefs as what influenced the decision of child marriage. The parents justified their decision of child marriage under religious beliefs. In addition, we revealed a more specific belief; sex outside wedlock as a religious taboo, and abortion is forbidden by religion.

#### Religion as a justification for child marriage

Several studies reported that child marriage was favored, based on the adherence to religious beliefs. As mothers and grandmothers taught their daughters and granddaughters that marriage was a prophet’s recommendation in Islam, an adolescent girl getting married at a young age was perceived as a good thing: “My mother and grandmother advised me to marry as soon as possible because marriage is one of our prophet’s recommendations” [38]. In Islam, based on religious doctrines, it is taught that when a person gets married, he/she indeed perfects half of his/her religion. This suggests that, regardless of their age, marriage is perceived as promoting human spiritual maturity in this religion. Concurrently, according to the Islamic religion, marriage should not be imposed if a girl is not ready. However, readiness for marriage is a subjective judgement that cannot be easily assessed by others, and as a consequence, child marriage is tolerated most of the time. When there were cases where the girls would insist on not being able to make decisions independently due to being too young and lack of sufficient knowledge and skills, the parents would still encourage their children to get married if there was a good proposal, as influenced by their religious beliefs.

#### Sex outside wedlock as a religious taboo

Several studies reported that sexual relationships outside wedlock are considered as a religious taboo among Muslim communities. Because of the religious beliefs, in Islamic countries, the parents favor the decision of early marriage for their daughters when they find out that their daughters are engaging in premarital sex. Due to the fear of pregnancy outside wedlock, the parents quickly marry off their daughters at the first opportunity: “My family believed that marriage can protect me from sin [sex outside marriage]” [38]. Further, from the girls’ perspective, due to the existing religious beliefs that having a baby outside marriage is a religious taboo, the adolescent mothers prior to marriage perceive having a child as the reason for receiving God’s blessing for their early marriage. By getting married as soon as they found out that a girl was impregnated, the family believe that they could avoid committing a sin.

*Abortion is forbidden by religion*. In a study conducted in Lebanon, there were few women who wanted to interrupt their pregnancies but were unable to find abortion services, as abortion is against local religious practices [29]. Unmarried pregnant women were left with no choice but to deliver the baby, and then forced into getting married at a young age: “No, it is forbidden (to abort). When a woman is pregnant, that’s it. There is nothing she could do.” [29].

### Theme 5: Individual circumstances, beliefs, and knowledge

Among the 12 articles, individual circumstances, beliefs, and knowledge emerged as facilitators of child marriage from seven studies [33–38, 40]. The participants (women (married and unmarried), refugees (married, divorced or formally engaged), and stakeholders (religious leaders, teachers, mothers, academics, healthcare workers, police, lawyers, government and NGO representatives)) described their personal beliefs and circumstances that favored child marriage.

#### Feelings of loneliness

Loneliness was one of the motivating factors for child marriage. Due to feelings of loneliness, the young female adolescents were encouraged to get married at an early age. The feelings of loneliness stem from the experience of separation from family, friends, school, and other events in their lives: “As I have no sister or brother, father, mother or friends, I decided to get pregnant, no one was beside me” [30]. The loneliness was further exacerbated in conflict settings when adolescent girls were living in a new neighborhood in the settlements and often no longer attending school after the conflict, which created a crude and lonely situation for them in the settlements. After the conflict, the girls could no longer focus on their studies. As a result of the conflict, family homes were destroyed and the whole family lacked livelihoods. This led the parents to seek better ways of life by forcing their daughters into early marriages. In these cases, child marriage was a coping strategy in the face of loneliness. The girl also had the desire to become pregnant, as they believed that having children would get rid of loneliness: “I like kids very much. I wanted to have children. When I saw other people’s children, I wanted to have a baby too” [30]. They believe that by having a baby, they would be able to fill the void in their lives.

#### Lack of autonomy in decision making

In Iran, Montazeri et al. interviewed women who visited health care centers in Ahvaz to understand the determinants of early marriage [38], and Mardi et al. investigated the perception of marriage among married teenage girls in Ardabil [35]. In a separate study, Knox interviewed married Palestinian adolescent girls in post-conflict settings in Lebanon to explore the decision making processes leading to early marriage [37]. “Low autonomy in decision-making,” namely “inappropriate decision-making skills,” “inadequate problem-solving skills,” “insufficient negotiation skills,” and “lack of critical-thinking skills,” were identified as contributing factors of early marriage influenced by psychosocial motivation. Participants believed that since they are young and immature, their decision-making and critical-thinking skills are limited, coupled with the lack of information on marriage and a reliable source of consultation, hence, arbitrarily relying on their parents to make decisions for them with regards to getting married. In wanting to become mature, some participants believed that marriage would enable them to be more mature and responsible. Although there were some participants who wished to delay marriage because of educational and career aspirations, the lack of negotiation skills resulted in them not being able to discuss their wishes with their parents, and eventually, being urged into getting married at an early age: “When my family suggested to get married, I cried all day because I was so young. I told my mother I don’t like to marry now. I was at first grade of high school at that time; I wanted to finish my education before getting married. But my parents were older than me and I could not convince them to accept my explanations” [38]. The theme of insufficient decision-making power was also mentioned in the study by Mardi et al. that analyzed how teenage women who got married at an early age describe their life after marriage, that the girls are deprived of their independence by their husband’s or family in law’s control even after the marriage: “…Marital life is very hard; cleaning the house, cooking, taking care of the baby, it is just unbearable. Sometimes I wish I was not married…” [35]. The subtlety of the girls’ emotions concerning autonomy was described by Knox in the sense that although the previous studies emphasize the lack of decision-making skills among the girls due to their parents’ strong influence, the married adolescent girls express that their decision to get married was not forcefully influenced by anyone, including their parents. From the girls’ perspective, they accepted and agreed to get married at an early age in consideration of their parents’ wishes: “I only agreed for one reason. I agreed because of the situation here, because I wanted to reduce the burden on my parents, because their economic situation is not good” [37]. Apart from skills, there are also desires and feelings. The participants described “social needs,” “emotional needs,” and “sexual needs” as other reasons for early marriage. On “social needs,” some viewed marriage as a route for a better living status/conditions, being able to gain respect from the husband’s family, to achieve peace of mind by escaping their own family’s stressful poor financial situation, and gaining independence from their own family. On “emotional needs” and “sexual needs,” the participants anticipated the “feeling of being loved” and believed that marriage was a way to satisfy their sexual needs to prevent premarital sex.

### Theme 6: Social norms

The theme of social norms appeared in 11 of the 12 studies [29–34, 36–38, 40, 41]. The participants (women (married and unmarried), refugee women (married and unmarried), mothers who experienced child marriage, their parents, and stakeholders (NGO and government officials, camp leader, religious leaders, teachers, academics, healthcare workers, lawyers, police and gynecologist)) often referred to social norm as what is influencing child marriage. In relation to child marriage, there were rigid social norms that were part of the participants’ community tradition and culture as included in the studies. Aside from the family values that were elaborated in a previous theme, social values are widely embraced in the whole community, which made it natural for them to accept child marriage.

#### Influence of patriarchal ideology

Some studies highlighted the issue of patriarchal ideology which influenced the decision of child marriage. This is a historically rooted problem, and such ideology is widely accepted among communities in Iran and Morocco as it functions as a powerful influence that places women in a submissive position to men [30, 33, 34, 38]. Because of this ideology, adolescent girls are satisfied by the decision of getting married at an early age as they submissively follow the decision made by their parents or the elder member of the family. The girls live in a society where gender and age discrimination prevail, thus the structures of the family and the society are stabilized by maintaining patriarchal ideology. In such a society, the voices of the elder member of the family are followed, in the name of respect. If a woman opposed her father’ order, she may face intimidation and ostracism from their families. It was reported that the authorities in charge of approving marriages, such as family court judges in Morocco, overruled the law and permitted child marriages, based on a patriarchal vision: “We deal with girls as young as 14, which is well below the minimum age stipulated in the Moudawana. Often there is an element of deceit: when girls look much older than their age, the judges don’t blink an eye” [34].

#### Threat to the social order and social protection

In Morocco, Iran, and Afghanistan, an unmarried status was considered as a threat to the social order for women and men alike [30, 33, 34, 36, 38]. Therefore, marriage is encouraged regardless of age. This social norm exists because of the strong belief that the institution of marriage is important in maintaining sexual relations only within marriage. Sex outside marriage is illegal in such countries. It is strongly believed that if such illegal acts occur, there would be risk and chaos in the society. Similarly, in Lebanon, there was a strong belief among the Syrian refugees in the country that child marriage would bring social protection to the girls and lead to the preservation of a girl’s honor in a conflict setting. This concept was called “al Sutra” among the Syrian refugees in Lebanon, and this social norm was widely shared among parents of the girls as well as the community [31]. Keeping the girls’ and the family’ honor is extremely important that the girls and their family highly value maintaining the girl’s virginity until she gets married. However, in a conflict setting, there are usually unfavorable rumors about a girl’s chastity, and often a focus on sexual harassment by men, due to their vulnerability as refugees: “According to this research, war and displacement increased refugees’ sense of insecurity and vulnerability and their real and perceived risks of sexual harassment. Thus, they felt an increased need to protect their daughters and their family’s honor, and many parents opted for child marriage as a means of doing so” [31]. In Afghanistan, among the religious leaders, it was believed that child marriage can protect the girls from illegal behaviors such as premarital sex and prostitution; therefore, if girls get married early, the risk can be reduced: “Girls increase economic status via marriage. If no school [i.e., if they have received no education] and no marry, they may turn to prostitution” [36].

#### Engagement of children

In some societies in the rural areas, there is a traditional practice of getting a child engaged at birth or during infancy. This is called “bad” or “badal” in Afghanistan, which refers to exchange of a child between the families or the communities in order to resolve conflict between them: “In our culture some girls are engaged at 2 years of age. Then her new family will wait for her for 8 or 9 more years; after that, they compel the family for marriage” (female NGO staff member) [36]. In this cultural context, the girls are being traded between the families in order to settle the disputes or to return the financial debts owed to the family to which they allow child engagement to. This is a deeply rooted traditional practice in Afghanistan, especially in the rural areas.

## Discussion

To the best of our knowledge, this is the first study where meta-synthesis of the factors that influence child marriage has been performed. We synthesized the findings from 12 qualitative studies. Interviews were conducted among women who were married before the age of 18, as well as among stakeholders such as healthcare professionals, NGOs, and so forth. The majority of the studies were from developing countries in the Middle East (Iran and Lebanon), Africa (Ethiopia, Morocco, and Nigeria), and South Asia (Afghanistan and Pakistan). The factors contributing to child marriage are complex and may vary across countries and regions.

We highlighted 6 themes and 21 categories. Closely linked and universally addressed themes that were underscored in our findings were: “Family Values and Circumstances,” “Religious Beliefs,” “Individual Circumstances, Beliefs, and Knowledge,” and “Social Norms.” We also found that themes such as “Legal issues” and “Human Insecurity and Conflict” are relatively under-addressed. However, they are gaining more attention due to recent increasing concerns about laws regarding marriage at a young age and conflicts worldwide.

“Family Values and Circumstances” seems to be an assortment of poverty and family ideologies that are in part shaped by cultural norms. In many impoverished contexts, marrying off the daughter at an early age is perceived as a strategy for economic survival for the family, and for the girl. Circumstances, in particular, family breakdown, divorce or death of a parent, often perpetuate financial constraints. Child marriage is also influenced by social norms and religious beliefs. In some regions, child marriage is believed to be the practice that preserves the family’s honor, to avoid the shame of having an unmarried daughter engaging in premarital sex or getting pregnant out of wedlock. Furthermore, in some societies, there is social pressure for women who remain unmarried for too long as their sexual purity is brought into question and this may jeopardize the family reputation [42]. In many societies under the Islamic religion, marriage is permissible as soon as the girl experiences her menarche. Concerning religious beliefs, marriage at a young age is perceived as a valuable event and even recommended based on moral, social, and psychological grounds. Specifically, when there are financial difficulties in the family, religious beliefs become a justification for the family to marry off their daughters at an early age. “Individual Circumstances, Beliefs, and Knowledge” factors, particularly lack of autonomy and negotiation skills, predisposed the girls to inevitably accept and obey their parents’ decision of their early marriage.

Globally, more countries are recognizing the harmful effects of child marriage, and are joining forces to scale-up legislations against this practice [43]. However, despite the awareness among families and communities about the legal minimum age for marriage, this practice continues due to underlying complex drivers. In some cases, social customs, religion, and traditional values are the justifications that outweighed the legal age restrictions of marriage [44]. This is in line with other reports that have identified legal gaps and inconsistent and limited enforcement of laws to prevent child marriage in many settings [23, 45–47]. When considering exceptions for the age of marriage with parental or judicial consent, close to 100 million girls around the world are not effectively legally protected against child marriage in their countries [47]. As suggested by Santelli et al., this is in part due to the indistinct legal systems in many countries [48]. Particularly, where child marriage is common, civil laws are often antagonistic with customary, religious, and common laws, complicating the legal compliance of frameworks against child marriage. Evidence has demonstrated that laws around the marriage age are inadequate and can drive the practice of child marriage underground, harming young people rather than protecting them. Countries need to invest in holistic approaches to tackle the underlying drivers of child marriage, by addressing poverty and gender inequality, and empowering young women through education and economic opportunities. What we can draw from the results of this meta-synthesis is that a holistic approach needs to be conducted by the experts from multiple disciplines in the fields of social welfare, law, economics, religion, education, gender studies, and public health by forming a multi-disciplinary team to work together toward a common goal of reducing child marriage.

Our study also underlines “Human insecurity and conflict” as a driver of child marriage. Growing evidence shows that the rate of child marriage is increasing at an alarming rate in humanitarian setting; nine out of the 10 countries with the highest child marriage rates are considered as either fragile or extremely fragile states [49]. Seven out of the 20 countries with the highest child marriage rates are countries with the biggest humanitarian crises. In our review, the included studies did not clearly describe the condition or type of humanitarian settings; situations can range from before, during, and after natural disasters, conflicts, and epidemics. These conditions aggravate poverty, insecurity, and truncated education, all of which are drivers of child marriage. Parents view child marriage as a way of coping with the worsening economic hardship, and of protecting girls from increased violence or rape. The number of civil wars has doubled since 2001, jumping from 30 to 70, the number of people killed in these armed conflicts has increased tenfold since 2005, and there are more refugees and internally displaced people around the world than at any time since the Second World War [50]. Nevertheless, child marriage is not being adequately addressed in humanitarian settings. More research is needed to understand child marriage in different crisis contexts, and to tailor solutions accordingly.

There are mixed results of what works in halting child marriage. Kalamar et al. assessed high-quality interventions and evaluations to reduce child marriage in low- and middle-income countries [51]. In their study, out of 11 included studies, six had positive results in terms of decreasing the proportion of married children or increasing the age at marriage, one had both positive and negative findings, and four had no statistical impact on the proportion of married children or age at marriage. The interventions that had statistical impact did not focus directly on child marriage (i.e., they had other targets such as reducing HIV/AIDS or improving sexual and reproductive health) or were closely related to structural factors such as schooling. Chae and Ngo reviewed 22 interventions to reduce the prevalence of child marriage and/or age at first sexual encounter across 13 low-and middle-income countries [52]. The most common approach was empowerment, followed by economic, schooling, and community approaches. Eleven studies were considered successful, six had mixed success, and five were unsuccessful. The most successful approach was empowerment such as life-skills and vocational-skills training, gender-rights awareness training as well as provision of sexual and reproductive health education and services, while the economic approach was the least successful. The authors recommended incorporating an empowerment approach, either as the sole approach or in conjunction with another approach for a higher success rate in reducing child marriage.

It is vital to address the interplay of each of the themes found in this study. For this viewpoint, we refer to the theories of family change and cultural tightness-looseness as the conceptual frameworks. Theory of family change highlighted that family act as a mediator of a young girl’s psychological development, as she grows up within a society and a culture [5]. In addition, in cultural tightness-looseness theory, values based on culture and social norms becomes the basis for judgement whether sanctions should be imposed on people in a particular society [6]. These theories provide us with the insights, when applied to the results of this study, that a young girl’s decision of child marriage is influenced by the values of her family, and also the social pressure that she senses within the society and culture in which she lives in. In this study, among the six themes, it was established that the theme of “individual circumstances, beliefs, and knowledge” was shaped by three themes; “family values and circumstances,” “religious beliefs,” and “social norms”. In addition, in a wider scope, there were two themes of “human insecurity and conflict,” and “legal issues,” that surround and accrue upon some of the participants without option, depending on the socio-political situation of the countries in which they live. Furthermore, in the complexity of the dynamic interplay of various factors, it is also important to highlight that the themes of “human insecurity and conflict” and “legal issues” are also closely linked with socio-cultural and religious aspects, such as religious beliefs, social norms, and family values. Our findings are endorsed by these theories that the factors that influence child marriage are in effect at multiple level, so that we need to take into considerations of all factors when coming up with the strategies to reduce child marriage. It is important to conduct further studies to analyse how and to which extent the themes found in this study interplay to synergistically influence child marriage.

## Conclusion and implications

This meta-synthesis described the factors that influence child marriage. The factors are intertwined and interventions will not work if they fail to account for all. There have been some encouraging examples as described above. Social change would require multisectoral collaboration by various stakeholders, empowering the girls themselves, their families, and communities. The response must also comprise a combination of concrete actions that lead to social changes.

From the results of this meta-synthesis, we also pointed out the lack of diverse studies in terms of income level, cultural and religious background, and geographical region. Most of the qualitative studies were from developing countries in Africa, the Middle East, and South Asia, lacking evidence from other parts of the world, such as Latin America and Southeast Asia, where the rates of adolescent pregnancies are the highest in the world [4]. The lack of studies investigating the influence of religion other than Islam on child marriage is a limitation in our meta-synthesis. Therefore, our results may overemphasize the influence of Islam on child marriage. Ending child marriage will not only contribute to the Sustainable Development Goals (SDGs) target 5.3 “Eliminate harmful practices such as child, early and forced marriage…,” but also to many other SDG targets including, but not limited to, eradicating extreme poverty (target 1.1), reducing maternal mortality (target 3.1), elimination of gender disparities in education (target 4.5), ending violence against all women and girls (target 5.2), universal access to sexual and reproductive health and rights (target 5.6), and ending all forms of violence against children (target 16.2) [19, 53]. The findings of this study identified factors that influence child marriage which are important to take into consideration when implementing programs to reduce child marriage. Eliminating these harmful practices is included in the SDGs target 5.3 aiming to tackle gender inequality. But our study also provides evidence that addressing poverty and conflict (SDGs Goals 1 and 16) can be critical to address child marriage. Thus, concrete measures must be taken to reduce child marriage globally by understanding the complexity and interrelationship among various targets.

In practice, based on the findings of this study, it is important to introduce programs that can improve the enforcement of laws related to child marriage. As we observed, legal frameworks related to child marriage were insufficiently enforced in many settings. Therefore, it is imperative that interventions related to legal issues that prevent child marriage are adequately enforced. Additionally, this study highlighted human insecurity and conflict as factors that affected child marriage. Thus, it is important to include child marriage prevention components in the intervention programs in refugee camps and resettlements in conflict settings.

## Supporting information

S1 File The ENTREQ checklist. (PDF)
