# Supplementary material for: Investigation of the key factors that influence the girls to enter into child marriage: A meta-synthesis of qualitative evidence
Source: PLoS One. 2020 Jul 17;15(7):e0235959. doi: 10.1371/journal.pone.0235959 (PMC7367464; doi:10.1371/journal.pone.0235959)
Supplement: S1 File — (PDF) [file pone.0235959.s001.pdf]

The ENTREQ Checklist

Title: Investigation of the key factors that influence the girls to enter into child marriage: A meta-synthesis of qualitative evidence

No.	Item	Guide and description	Reported on page #
1	Aim	State the research question the synthesis addresses:<br>*To identify key factors influencing child marriage and synthesize findings from qualitative studies.*	5-6
2	Synthesis Methodology	Identify the synthesis methodology or theoretical framework which underpins the synthesis, and describe the rationale for choice of methodology (e.g. meta-ethnography, thematic synthesis, critical interpretive synthesis, grounded theory synthesis, realist synthesis, meta-aggregation, meta-study, framework synthesis)	6, 8-9 (Methods)
3	Approach to Searching	Indicate whether the search was pre-planned (comprehensive search strategies to seek all available studies) or iterative (to seek all available concepts until they theoretical saturation is achieved):<br>*The search was pre-planned. Comprehensive search strategies were undertaken to seek all available studies.*	6-7 (Methods)
4	Inclusion Criteria	Specify the inclusion/exclusion criteria (e.g. in terms of population, language, year limits, type of publication, study type):<br>*Included studies used widely accepted qualitative data collection methods, with well-described methodology, including for example: interviews, focus groups, and direct observation. Included studies also needed to have provided a clear description of recognized qualitative data analysis methods (e.g., grounded theory, narrative analysis, content analysis, thematic analysis). Excluded studies included those for which it was difficult to extract qualitative data, e.g., mixed methods studies.*	6-7 (Methods)
5	Data Sources	Describe the information sources used (e.g. electronic databases (MEDLINE, EMBASE, CINAHL, psycINFO, Econlit),	7-8 (Methods)

		grey literature databases (digital thesis, policy reports), relevant organisational websites, experts, information specialists, generic web searches (Google Scholar) hand searching, reference lists) and when the searches conducted; provide the rationale for using the data sources: *The following electronic databases were considered to be the most relevant for the topic and were searched: PubMed, PsycINFO, CINAHL, Scopus, ProQuest, Web of Science Core Collection, ScienceDirect, Kyoto University Discovery and Google Scholar. All data were limited to publications in English from 2008 to 2018.*	
6	Electronic Search Strategy	Describe the literature search (e.g. provide electronic search strategies with population terms, clinical or health topic terms, experiential or social phenomena related terms, filters for qualitative research, and search limits): *Key search words included: "women", "girl", "young*", "teen", "mother", "child marriage", "early marriage", "teenage marriage", "health", "interview", "focus group*", "case stud*", "observ*", "view*", "experience*", "opinion*", "attitude*", "percep*", "belie*", "feel*", "know*", "understand*", "qualitative". We combined key words applying the Boolean operators AND/OR.*	7-8 (Methods)
7	Study Screening Methods	Describe the process of study screening and sifting (e.g. title, abstract and full text review, number of independent reviewers who screened studies): *We screened all titles and abstracts of the initial 3,859 "hits" and after eliminating duplicates and those not satisfying the inclusion criteria (3,835 articles) we filtered 24 studies as potentially eligible. We checked their reference lists and identified 11 further studies. Upon reading the articles in full text, we finally selected 12 studies for our analysis. Fig 1 shows the overall process.*	7-8 (Methods)
8	Study Characteristics	Present the characteristics of the included studies (e.g. year of publication, country, population, number of participants, data collection, methodology, analysis, research questions):	Table 2

		Table 2 presents the characteristics of the included studies (author(s), year of publication, country, population, description of the participants, age of women at the time of study, age at marriage, sample size, research design, and data collection method.	
9	Study Selection Results	Identify the number of studies screened and provide reasons for study exclusion (e,g, for comprehensive searching, provide numbers of studies screened and reasons for exclusion indicated in a figure/flowchart; for iterative searching describe reasons for study exclusion and inclusion based on modifications to the research question and/or contribution to theory development):<br>*A flow diagram using PRISMA guidelines for reporting of systematic reviews is presented in Figure 1 in reporting of the selection process and results. We identified 3,859 studies after removing duplicates and excluded Records excluded 3,835 studies by reviewing the title and abstract. As a result, we included 24 studies after initial screening by title and abstract. After that, we have read the papers in full text and further excluded 13 studies as they did not fit to the aim of the study. In addition, we have checked the references of the 24 studies at the initial stage, and found 11 studies that were potentially eligible, but excluded 10 studies after reading the texts in full. After going through these processes, we included 12 studies to be analyzed in this study.*	8, Fig. 1
10	Rationale for Appraisal	Describe the rationale and approach used to appraise the included studies or selected findings (e.g. assessment of conduct (validity and robustness), assessment of reporting (transparency), assessment of content and utility of the findings):<br>*Critical Appraisal Skills Program (CASP) guidelines were used to assess qualitative evidence syntheses findings. CASP offers a valid checklist to help researchers appraise and understand qualitative studies. In order to come up with a score for CASP check, a three-point rating*	8-9, Table 1

		system developed by Duggleby and colleagues was adopted in this study.	
11	Appraisal Items	State the tools, frameworks and criteria used to appraise the studies or selected findings (e.g. Existing tools: CASP, QARI, COREQ, Mays and Pope [25]; reviewer developed tools; describe the domains assessed: research team, study design, data analysis and interpretations, reporting):<br>*CASP guidelines was used to assess qualitative evidence of syntheses findings.*	8-9
12	Appraisal Process	Indicate whether the appraisal was conducted independently by more than one reviewer and if consensus was required:<br>*Three researchers (AK, TT, and SPS) independently read, reread, and appraised the articles. Three-point rating system developed by Duggleby and colleagues was adopted in this study to quantify the appraisal from the CASP checklist and obtain an overall score of quality.*	8-9
13	Appraisal Results	Present results of the quality assessment and indicate which articles, if any, were weighted/excluded based on the assessment and give the rationale:<br>*Appraisal results are presented in Tables 1.*	Table 1
14	Data Extraction	Indicate which sections of the primary studies were analysed and how were the data extracted from the primary studies? (e.g. all text under the headings "results /conclusions" were extracted electronically and entered into a computer software):<br>*Analysis of the synthesized studies followed the steps of thematic synthesis. In doing so, we categorized the codes into three groups: "first-order" theme representing the perspectives and understanding of the participants of the studies; "second-order" theme representing the primary authors' interpretations; and "third-order" themes reflecting the interpretation of the researchers.*	9-10
15	Software	State the computer software used, if any:<br>*Microsoft Excel software was used for*	10

		data management and analysis.	
16	Number of Reviewers	Identify who was involved in coding and analysis:<br>*Three reviewers were involved in coding and analysis.*	9-10
17	Coding	Describe the process for coding of data (e.g. line by line coding to search for concepts):<br>*Thematic analysis was employed to identify factors influencing child marriage.*	9-10
18	Study Comparison	Describe how were comparisons made within and across studies (e.g. subsequent studies were coded into pre-existing concepts, and new concepts were created when deemed necessary):<br>*Similar findings were coded into key themes and categories within and across studies.*	9-10
19	Derivation of Themes	Explain whether the process of deriving the themes or constructs was inductive or deductive:<br>*The text from the results and discussion sections of the included articles were coded inductively and individually "line-by-line" to derive initial codes.*	9-10 (Inductive)
20	Quotations	Provide quotations from the primary studies to illustrate themes/constructs, and identify whether the quotations were participant quotations of the author's interpretation:<br>*Words used to explain in the included studies were referred in explanation of each theme in the form of brief quotes but not the segments of the included studies were used in this study as quotations.*	11-15, 17-27 (Many quotes throughout these pages)
21	Synthesis Output	Present rich, compelling and useful results that go beyond a summary of the primary studies (e.g. new interpretation, models of evidence, conceptual models, analytical framework, development of a new theory or construct):<br>*Synthesis output is presented in Table 3 in the form of metasummary.*	10-27 (Results section), Table 3
